# What Does the Anatomical Organization of the Entorhinal Cortex Tell Us?

**DOI:** 10.1155/2008/381243

**Published:** 2008-08-28

**Authors:** Cathrin B. Canto, Floris G. Wouterlood, Menno P. Witter

**Affiliations:** ^1^Department of Neuroscience, Kavli Institute for Systems Neuroscience and Centre for the Biology of Memory, Norwegian University of Science and Technology (NTNU), Building MTFS, 7489 Trondheim, Norway; ^2^Department of Anatomy and Neurosciences, Institute for Clinical and Experimental Neurosciences, VU University Medical Center, Amsterdam, P.O. Box 7057, 1007MB Amsterdam, The Netherlands

## Abstract

The entorhinal cortex is commonly perceived as a major input and output structure of the hippocampal formation, entertaining the role of the nodal point of cortico-hippocampal circuits. Superficial layers receive convergent cortical information, which is relayed to structures in the hippocampus, and hippocampal output reaches deep layers of entorhinal cortex, that project back to the cortex. The finding of the grid cells in all layers and reports on interactions between deep and superficial layers indicate that this rather simplistic perception may be at fault. Therefore, an integrative approach on the entorhinal cortex, that takes into account recent additions to our knowledge database on entorhinal connectivity, is timely. We argue that layers in entorhinal cortex show different functional characteristics most likely not on the basis of strikingly different inputs or outputs, but much more likely on the basis of differences in intrinsic organization, combined with very specific sets of inputs. Here, we aim to summarize recent anatomical data supporting the notion that the traditional description of the entorhinal cortex as a layered input-output structure for the hippocampal formation does not give the deserved credit to what this structure might be contributing to the overall functions of cortico-hippocampal networks.

## 1. INTRODUCTION

The entorhinal cortex (Brodman area 28) derives its name from the fact that it is partially enclosed by the rhinal (olfactory) sulcus. This feature is particularly striking in nonprimate mammalian species, but also in primates at least the anterior part of the entorhinal cortex is bordered laterally by a rhinal sulcus. Interest in the entorhinal cortex arose around the turn of the 20th century when Ramon y Cajal, in his seminal studies on the anatomy of the nervous system, described a peculiar part of the posterior temporal cortex which is strongly connected to the hippocampus with fibers that merge in the angular bundle and perforate the subiculum. Cajal was so struck by this massive connection that he suggested that the physiological significance of the hippocampus had to be related to that of the entorhinal cortex. At that time, he assumed that the entorhinal cortex was part of the olfactory cortex and so was, therefore, the hippocampus. He even stated that if this part of the posterior temporal cortex, which he called the sphenoidal cortex/angular ganglion, would be visual, so would be the hippocampus [1]. How right he was, in more than one way. Today we conceive the entorhinal cortex as the nodal point between the hippocampal formation on the one hand and a variety of multimodal association areas of the cortex such as parietal, temporal, and prefrontal cortex on the other hand. So, multimodal sensory and highly processed unimodal inputs converge at the level of the entorhinal cortex. This input in turn is conveyed to the hippocampal formation. We also know that the entorhinal cortex harbors different subdivisions, which specifically mediate the connectivity with functionally different sets of cortical and subcortical areas in the brain. This has led to the now quite widely accepted concept of parallel input/output channels as originally proposed by us and others [2–5]. Recent electrophysiological recordings in lateral and medial entorhinal cortices of the rat have further elaborated on this point in showing that cells in the medial subdivision are spatially modulated, whereas in the lateral entorhinal cortex such modulation is largely absent [6–9]. Cells in the lateral entorhinal cortex most likely convey olfactory [5, 10, 11] and somatosensory information [12–15].

Our current insights into the functional relevance of the hippocampal formation, and how its anatomy is related to function, are much more detailed than what we know about the entorhinal cortex. It therefore seems attractive to turn the argument of Cajal around by stating that in view of the findings that the hippocampus is crucially involved in conscious, declarative memory processes so should be the entorhinal cortex. This conjecture is apparently supported by available functional studies. Although the specific functional contributions of the entorhinal cortex to memory remain to be established, they are most likely different from, but complementary to, those of the hippocampus [5, 16, 17]. The finding of the grid cells in medial entorhinal cortex, as well as head direction and conjunctive cells, and the notion that converging inputs of a limited number of grid cells onto a single CA1 neuron are sufficient to result in the well-established place cell properties [17–19] kindled a renewed interest in the anatomical organization of the entorhinal cortex [20]. A review of the anatomical organization, as part of a special issue on entorhinal cortex, is therefore appropriate. We aim to provide a comprehensive description of the entorhinal cortex, with particular emphasis on the intrinsic organization, based on data from studies in the rat, extensively referring to, rather than repeating, previously published accounts.

## 2. DEFINITION OF THE ENTORHINAL CORTEX, SUBDIVISIONS, AND OVERALL ARCHITECTURE

A cortical area can be defined in many different ways, using a variety of different criteria, such as location, connectivity, cyto- and chemoarchitectonics. For the entorhinal cortex, all these approaches have been applied, resulting in a confusing variety of borders, subdivisions, and description of layers. A good lead, since it has withstood over a century of arguments, is the definition of the entorhinal cortex on the basis of its connectivity with the hippocampus as originally suggested by Cajal [1]. In view of increasing insights into the connectivity of the hippocampal formation and its subdivisions, quite a few authors have chosen to take projections to the dentate gyrus as a good defining criterion, in particular in combination with certain cytoarchitectonic features. In this paper, such a combined definition will be used and described below.

The entorhinal cortex is surrounded by a number of cortical areas. Anteriorly, it meets with olfactory and amygdaloid cortices, such as the piriform (olfactory) cortex laterally, and medially it is bordered by the periamygdaloid cortex and the posterior cortical nucleus of the amygdala. On its medial side, the entorhinal cortex merges with structures that belong either to the hippocampal formation or the parahippocampal region, such as the amygdalo-hippocampal transition, and the parasubiculum. The lateral and posterior borders are with the other two major constituents of the parahippocampal region, the perirhinal cortex laterally and the parahippocampal cortex (in nonprimate species generally referred to as postrhinal cortex) posteriorly. The lateral and posterior borders are quite easy to establish on the basis of a variety of cytoarchitectonic and chemoarchitectonic features. The most prominent features are that the fairly large-sized cells of layer II in the entorhinal cortex are replaced by much smaller neurons in the perirhinal and postrhinal cortices, the lamina dissecans disappears, and these changes coincide with similarly striking changes in the density of parvalbumin-positive neuropil, high in entorhinal cortex, virtually absent in perirhinal and parahippocampal areas. The mirror-image pattern appears when staining for heavy metals (Timm stain) or the calcium binding protein calbindin. All additional criteria that have been described seem to coincide with these borders. The anterior and medial borders, in contrast, are somewhat harder to establish. They apparently coincide with a rather striking change in the ease with which layers II and III can be differentiated from each other as well as with a loss of differentiation between the deep layers (medial border) or even complete disappearance of the deep layers (anterior border). Combined with subtle changes in chemoarchitectonic features and connectional differences, an overall consensus has now been reached (for further details see [21–25]).

Attempts to subdivide the entorhinal cortex have, likewise, been numerous (see [26], for a detailed review cf. [27]). Whereas Cajal, similar to Lorente de Nó did not see much merit to subdivide the entorhinal cortex based on cytoarchitectonic criteria [28, 29], it was Brodmann [30] who parcelled the entorhinal cortex field 28 into two fields: a lateral area 28a, and a medial area 28b on the basis of cytoarchitectonic criteria. Lorente de Nó [29] instead argued that the projections to the hippocampal formation support to distinguish between lateral, intermediate, and medial entorhinal subdivisions. The use of these two fundamentally different approaches, connectivity versus architecture, has continued till today, although a merged approach is now becoming accepted. Cytoarchitectonic parcellation schemes are useful tools to describe experimental data about connectivity and data on for example the distribution of receptors [2, 22, 31–33]; they help to navigate through data. Connectionally based subdivision-schemes may better serve our understanding of the possible functional contributions [34]. In view of the strong implications of the human entorhinal cortex in a variety of brain diseases (see, e.g., [35, 36]), the development of animal models for such diseases depends strongly on our capabilities to extrapolate the definition of the entorhinal cortex from rodents to nonhuman and human primates. With this aim in mind, combinations of the different approaches may lead to the most detailed and reliable subdivision.

A good start to subdivide the entorhinal cortex is to use the entorhinal-to-dentate projection, which has been documented in extensive detail in a variety of species. On the basis of the terminal distribution of this projection in the rat and the mouse, it seems plausible to divide the entorhinal cortex into two subareas, generally referred to as the lateral and medial entorhinal cortices (LEC and MEC, resp.). These areas roughly correspond to the description of Brodmann's areas 28a and b, respectively [25, 30, 37, 38]. In the monkey [39], the terminal distribution of the entorhinal-to-dentate projection does not provide such a clear criterion to functionally subdivide the entorhinal cortex. However, a second connection, which has been proposed to functionally subdivide the entorhinal cortex, is the input of the presubiculum. In all nonprimate mammalian species studied so far, including rat, guinea pig, and cat, the innervation of the entorhinal cortex by presubicular fibers is restricted to a more caudal and dorsal portion, that coincides with a cyto- and chemoarchitectonically well-defined area, now called MEC [40–44]. Also in the monkey, inputs from the presubiculum distribute to only a restricted posterior portion of the entorhinal cortex ([45, 46]; Witter and Amaral, unpublished observations), which may thus represent the homologue of MEC as defined in nonprimates.

**Figure 1:**
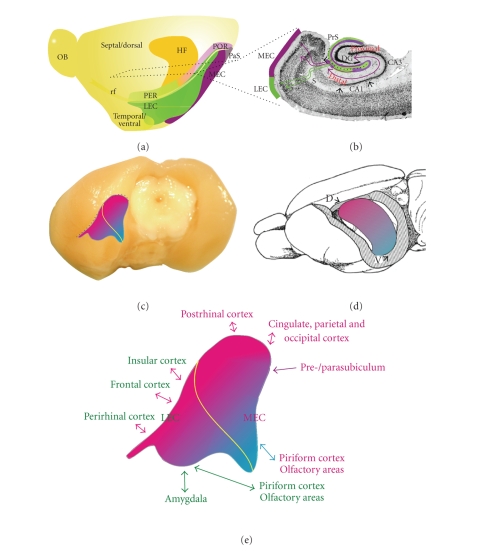
Schematic representation of the overall organization of the entorhinal cortex and its connectivity. (a) Position of the entorhinal cortex and surrounding cortices and hippocampus in the rat left hemisphere. Indicated are the dorsoventral extent of the hippocampus, positions of LEC and MEC, and the approximate position of a representative horizontal section, illustrated in (b). (b) Horizontal section illustrating entorhinal-hippocampal connectivity (see text for more details). (c) and (d) Representation of the topographical arrangement of entorhinal-hippocampal reciprocal connections. A dorsolateral band of entorhinal cortex (magenta) is preferentially connected to the dorsal hippocampus. Increasingly, more ventral and medial bands of entorhinal cortex (purple to blue) are connected to increasingly more ventral levels of the hippocampus. Yellow line in (c) indicates the border between LEC and MEC. (e) Enlarged entorhinal cortex, taken from (c), indicating the main connectivity of different portions of entorhinal cortex. Brain areas preferentially connected to LEC are printed in green, those connected to MEC are in magenta. The color of the arrows indicates preferential connectivity to the dorsolateral-toventromedial bands of entorhinal cortex (magenta or blue, resp.) or that no preferential gradient is present (green).

A note of caution should be added here: the choice for the terms lateral and medial entorhinal cortex is not simply related to a particular anatomical position of these areas in relation to the hippocampal formation and the rhinal fissure. In general, the lateral area occupies a more rostrolateral position versus a more caudomedial position for the medial area (see Figure 1(a)).

The lamination of the entorhinal cortex generally is considered the prototype of the transition between the three-layered allocortex and the six-layered neocortex [26]. The superficial plexiform or molecular layer (layer I) is relatively free of neurons and, in general, contains a dense band of transversely oriented fibers. The outermost cell layer (layer II) varies considerably in appearance among the rostro-to-caudal and lateral-to-medial extent, but mainly contains so-called “stellate” or “modified pyramidal cells.” Overall, cells in layer II are fairly large, making them distinctly different from layer II cells in the adjacent cortical regions with the exception of the parasubiculum. In the latter area, neurons of layer II are as large as or somewhat larger than those of the entorhinal cortex, but entorhinal cells stain darker with a Nissl stain. Layer III is a wide layer of loosely arranged, large to medium sized cells that are predominantly of the pyramidal type. The deep border of layer III is the cell-sparse fiber layer called the lamina dissecans (sometimes referred to as layer IV). The lamina dissecans is better developed in the medial entorhinal cortex although species differences are apparent. The next cell layer (layer V) is clearly stratified and sometimes subdivided into a superficial layer of large to medium-sized, darkly stained pyramidal cells, which is sometimes referred to as layer Va. Note that in some lamination schemes, more particularly so in primates, this layer is referred to as layer IV thus resulting in some confusion when compared to the present scheme where the lamina dissecans is referred to layer IV. Subsequent deeper portions of layer V (layer Vb/Vc) have an overall stratified appearance and mainly consist of rather small pyramidal cells with a moderately dense packing. In the deepest cell layer VI, which is delineated by the white matter, multiple layers can be distinguished, more in particular in primates. However, since the appearance of layer VI is highly variable at different lateromedial and rostrocaudal levels, generally no further differentiation between sublayers is made.

## 3. EXTRINSIC CONNECTIVITY

### 3.1. Entorhinal hippocampal connectivity

Entorhinal connections with the hippocampal formation in the rat have been comprehensively described and reviewed in a number of recently published papers and reviews to which the reader is referred for further details [2, 47–52]. To summarize, all regions of the entorhinal cortex project to all parts of the hippocampal formation, the dentate gyrus, fields CA3, CA2, CA1, and the subiculum. Overall, entorhinal fibers synapse most often onto the dendrites of principal cells, that is, on spines, where they form asymmetrical, excitatory synapses. Entorhinal fibers also terminate on inhibitory interneurons, forming both putative excitatory as well as inhibitory synapses with the dendrites of these interneurons [38, 47, 52–54]. In the dentate gyrus, entorhinal axons distribute largely to the outer two-thirds of the molecular layer, although differences between species may exist with respect to the precise terminal distribution in relation to the origin of these projections in either LEC or MEC [38, 55]. The projections to the dentate gyrus arise largely from neurons in layer II. However, projections that arise from the deep layers have been systematically observed, and it is likely that these deep originating fibers show a differential terminal distribution, largely innervating the inner molecular layer of the dentate gyrus ([56]; see also [38]). The same cells in layer II also form the main origin of the projection that distributes to the outer portions of stratum lacunosum-moleculare of CA3 and CA2 [38, 49]. In all species studied, the projections to CA1 and the subiculum originate from cells in layer III of both LEC and MEC. The terminations of the latter projections exhibit a transverse topography. The rostral entorhinal cortex in the monkey and LEC in the rat project to the region around the border between CA1 and subiculum (distal CA1, *furthest away from the dentate gyrus*, and proximal subiculum, *closest to the dentate gyrus*) whereas caudal entorhinal cortex in the monkey and MEC in the rat project to proximal CA1 (*close to the dentate gyrus*) and distal subiculum (*far from the dentate gyrus*) [55, 57, 58].

The CA1-subicular projections are topographically organized along the transverse or proximodistal axis as well, such that parts of CA1 and subiculum that receive comparable inputs that are either from LEC or MEC are connected to each other [59–61]. Finally, the projections from CA1 and subiculum back to deep layers of LEC or MEC grossly reciprocate the forward projections [51, 62, 63] (see Figures 1(a), 1(b)). These data thus indicate that the entorhinal-CA1-subiculum circuitry exhibits a high degree of fidelity, which suggests that this circuitry may permit a highly ordered processing of information. The functional relevance of this strikingly precise organization needs yet to be established. In this respect, it is of interest that the CA1 and entorhinal projections targeting the same population of subicular neurons do not seem to have a high incidence of convergence [64]. In contrast, in CA1, inputs from CA3 and entorhinal cortex do converge on pyramidal cells as well as onto interneurons with a very high incidence [54]. A final point to make with respect to entorhinal-hippocampal connectivity has to do with the topographical organization along the long axis of the hippocampus. Although the orientation of the hippocampus in various species is quite different [2, 65] in all species the structure has an impressive length, measuring from about 7 mm in mice, through 9–11 mm in rats, up to 4.5–5 cm in humans. It has now been established that in all species studied, entorhinal-hippocampal connectivity is present as described above, the striking difference being that different portions along the long axis of the hippocampal formation are connected to different bands of the entorhinal cortex. These bands are differently distanced from the lateral and posterior borders of the entorhinal cortex with the adjacent perirhinal and postrhinal (rodent) or parahippocampal (primates) cortices (see Figures 1(c), 1(d)). In the rat, this longitudinal topography has been shown to be closely related to the spatial properties of neurons in both structures. Neurons in the dorsal hippocampus and the related dorsal portions of entorhinal cortex, more in particular of the medial entorhinal cortex, exhibit firing patterns that, although qualitatively as well as quantitatively very different, both represent fairly small areas of the environment. In contrast, cells that are more ventrally positioned in both hippocampus and entorhinal cortex show much larger spatially tuned firing fields [8, 9, 66]. Interestingly, the relationships in this respect between the dorsal to ventral axes both in the entorhinal cortex and the hippocampus are reflected in a comparable relationship with respect to the behavioral effects of selective lesions. Whereas lesions in the dorsal hippocampus and dorsal part of the entorhinal cortex have comparable detrimental effects on spatial learning and recall, ventral lesions do not. The latter have a profound effect in fear related behavior, which is in turn not effected by dorsal lesions [16, 67].

### 3.2. Entorhinal cortical connectivity

The most comprehensive systematic series of studies on entorhinal connectivity in the rat is from the Burwell lab [13–15, 68–71], with some added studies describing one or a few inputs or outputs in greater detail [22, 72]. All these studies are in line with earlier influential reports in the monkey that the perirhinal and parahippocampal cortices form the major cortical link for the entorhinal cortex [73, 74]. In general, the perirhinal cortex projects to rostrolateral parts of the entorhinal cortex, whereas the parahippocampal (primates) or postrhinal (nonprimates) cortex projects preferentially to caudodorsal portions of the entorhinal cortex [3, 68, 75]. In addition to the perirhinal/parahippocampal connections, the pre- and parasubiculum [23, 44] and olfactory-related structures provide prominent inputs to the entorhinal cortex and, in case of olfactory domains, receive a similarly strong output from the entorhinal cortex. As mentioned above, the spatial distribution of the input from the presubiculum is currently considered to be one of the defining features of MEC in different species. Though typically not as strong, additional cortical afferents and efferents of the entorhinal cortex are widespread. Cortical afferents are dominated by piriform input, but input also arises in frontal, cingular, retrosplenial, insular, parietal, and even visual areas. Similar to what was reported for the monkey, in the rat projections from the cingulate and retrosplenial cortices preferentially project to the more caudal portions of the lateral, intermediate, and medial bands of the entorhinal cortex [23, 72, 76–78]. Cortical efferents are widespread, largely reciprocating the cortical afferents. Note that species differences are apparent indicating that whereas in the rat cortico-entorhinal reciprocal connectivity is rather limited and confined to the areas close to the rhinal sulcus [22, 79], such connections in the monkey are more common and involve a more widespread domain of the entorhinal cortex [75, 80].

Although we will address the layered organization of the entorhinal cortex in more detail below, it is relevant to point out that entorhinal-cortical projections largely arise from deep layers, primarily from layer V pyramidal neurons. Possible exceptions are the entorhinal-infralimbic and entorhinal-olfactory projections, which appear to arise in layers II and III as well [22, 81]. Regarding entorhinal afferents, it is clear that most show a distribution largely confined to the superficial layers I–III with the exception of inputs from infralimbic, and prelimbic areas together with cingular and retrosplenial inputs that show a striking preference for deep layers of the entorhinal cortex [72].

### 3.3. Entorhinal subcortical connectivity

Studies conducted in multiple species indicate extensive subcortical connectivity for the entorhinal cortex. Although differences exist with respect to the detail of the information, it is safe to conclude that the entorhinal cortex has connections with the basal forebrain, claustrum, amygdala, basal ganglia, thalamus, hypothalamus, and brainstem (for review see [70]). The entorhinal cortex sends projections to the nucleus accumbens [82–85] and receives inputs from the ventral tegmental area [86]. The entire entorhinal cortex has strong reciprocal connections with the claustrum [32, 87–90]. Additional connections exist with basal forebrain structures, in particular the medial septal nucleus, the nucleus of the diagonal band, and the substantia innominata [32, 86, 91–93]. It is most likely that entorhinal projections to basal forebrain structures arise in layers II and V.

Entorhinal-amygdala connectivity has been studied in rather detail in both monkey and rat. For recent reviews, the reader is referred to McDonald [94], Pitkänen et al. [33]; see also Burwell and Witter [70]. Although all parts of the entorhinal cortex are connected with the amygdala, the rostral subfields are more strongly interconnected with the amygdala than the caudal subfields. Whereas in monkey the primary connections are with the lateral and accessory basal nuclei [95, 96], in rat the most prominent inputs arise from the lateral, basal, and accessory basal nuclei [97]. Amygdala input terminates primarily in layer III of the entorhinal cortex, and the return projection originates predominantly from cells in layer V.

The entorhinal cortex is connected with thalamic and hypothalamic structures. Major thalamic input arises in midline nuclei, particularly the reuniens, paratenial, and periventricular nuclei [31, 86, 98–100]. Additional but weaker inputs have been described from the anteromedial thalamic nucleus [101], and the ventromedial nucleus of the hypothalamus [102]. In the rat, it has been shown that the entorhinal cortex reciprocates the reuniens input [103]. In the monkey, additional projections have been reported to end in the magnocellular portion of dorsal medial nucleus, the medial pulvinar, and the dorsolateral nucleus [98, 104]. The entorhinal cortex also receives input from midbrain structures such as the dorsal raphe nucleus, the median raphe, and locus coeruleus [86, 105, 106]. Details about entorhinal innervations from these important modulatory regions of the brain are not available yet.

## 4. INTRINSIC ORGANIZATION OF THE ENTORHINAL CORTEX

Our understanding of the entorhinal cortex is still rather premature, and to a large extent, influenced by our current functional concept for MEC. The generally accepted division of the entorhinal cortex into at least two functionally different domains stresses the need for an answer to the questions whether or not they differ with respect to their intrinsic wiring and neuronal makeup, in addition to their gross differences with respect to cortical and subcortical connectivity summarized above. The entorhinal network, *grosso modo*, encompasses three different (groups of) elements, elements receiving inputs, elements that provide output, and elements that contribute to the intrinsic architecture of the area. This subdivision into three functionally different elements and roles to be played by different neurons does not necessarily have an exclusive character; it is actually quite likely that all three elements might be an integral part of one and the same neuron; however, specializations may occur.

**Figure 2:**
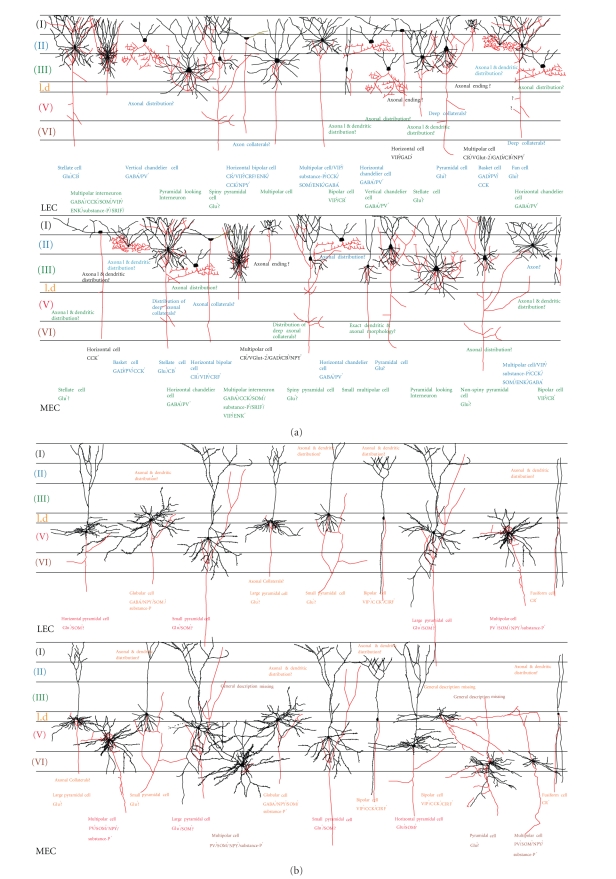
Summary diagram of the morphology of main cell types in LEC and MEC. (a) Cells in superficial layers I-III. (b) Cells in deep layers IV-VI. See text for more details.

Compared to the details known for the hippocampal formation and some parts of the neocortex, such as the visual or barrel cortices in rodents, our understanding of the entorhinal cortex is rather in its infancy. The first detailed description of the morphology of entorhinal neurons, based on Golgi impregnated material, was published in 1933 by Lorente de Nó [29]. Over the years, this initial description has been extended, adding details and new cell types, based on a variety of different techniques. Here, we will summarize the main cell types that are currently known with a focus on their local connectivity and in particular addressing the question whether or not the lateral and medial subdivisions differ with respect to the overall main cell types. We will summarize (see Figure 2) data from previously published reviews [107] supplemented with some recently obtained, yet unpublished own data.

### 4.1. Cell types in entorhinal cortex

**Layer I**

In layer I throughout the entorhinal cortex, Lorente de Nó described two cell types; horizontal cells and short axis cylinder cells, nowadays known as multipolar neurons (MPNs). This latter category constitutes the majority of cells in layer I, and generally, they are non- or sparsely spiny. MPNs are quite often positive for calretinin (CR) and GABAergic, and two types have been described. Small CR positive MPNs are more often located just deep to layer Ia [108], whereas CR positive MPNs with a laterally extending dendritic tree are mainly located deep in layer I [108]. From the perikaryon of MPNs three to five short, curved smooth dendrites arise that branch after a short distance and radiate within layer I, sometimes extending into layer II [108, 109]. The diameter of the dendritic tree is around 100 *μ*m in small or 150 *μ*m in the other MPNs, respectively. Own recent data indicate that the axons of layer I neurons travel towards layer II and III [110] where they most likely provide feed-forward inhibition to principal cells [111, 112]. A minority of CR positive layer I neurons can be glutamatergic or contain calbindin D28K (CB) or neuropeptide-Y (NPY) [113].

Horizontal cells are located in the transitional zone between layers I and II [29, 114, 115] . They have a spherical to elongated soma of 13–15 *μ*m. Almost spine-free dendrites extend laterally and spread horizontally within layer I and superficial layer II. The horizontal extent can be up to 700 *μ*m (own unpublished data). The noncollateralizing axon travels towards the deep layers to the hippocampus [110, 114, 115]. Horizontal neurons are GABAergic, in LEC some are positive for vasoactive intestinal polypeptide (VIP), whereas in MEC, the dendritic terminals can stain positive for cholecystokinin (CCK) [115–117].

**Layer II**

Layer II is mainly made up of densely packed, large and medium sized pyramidal and stellate cells. The most abundant cell type throughout layer II in MEC is the stellate cell, with their preferred location within superficial and middle layer II [118]. The soma of these cells is quite variable but their spiny dendritic tree is their defining characteristic. The dendritic arbor comprises multiple, roughly equally sized primary dendrites that branch widely (average extend of 497 ± 154 *μ*m) and may cover about one half of the mediolateral extend of the MEC [118–121]. After reaching the pial surface, the dendrites curve and run parallel to it. The basal dendritic extent is smaller (average of 231 ± 69 *μ*m) [118]. The relative thick axon of stellate cells courses straight towards the angular bundle from a primary dendrite or the base of the soma [120]. Up to 400 *μ*m away from the start, the axon gives off very thin collaterals, branching repeatedly and reaching the superficial layers, forming a net that colocalizes with the entire dendritic tree, sometimes extending beyond [118]. Besides, the axon sends occasional collaterals into deep layers III–VI. In the angular bundle, it gives off one to three collaterals that travel into the subiculum, continuing to their main targets in the dentate gyrus and CA3 [122]. Most stellate cells are excitatory presumably using glutamate as their main transmitter [123–126] and some also stain positively for CB [107].

Stellate cells are less common in LEC than in MEC. In LEC, stellate cells are most likely replaced by a comparable cell type, called fan cells [120, 127]. They have large polygonal somata with multiple thick sparsely spiny primary dendrites that fan out from the soma mostly in the horizontal and ascending direction. This dendritic morphology is thus comparable to that of stellate cells in MEC. The morphological difference is that fan cells only have small descending dendrites but there are also physiological differences. The axons descend and can be followed into the angular bundle, sometimes giving of very thin ascending collaterals within layers II and III [127].

Aside from the stellate-like principal neurons, layer II contains a number of pyramidal-like cells that have medium sized triangular or ovoid shaped soma with a perpendicular elongation with respect to the pial surface. Most are located in the deep portion of layer II [110, 128, 129]. The majority of these cells have a prominent spiny thick apical dendrite branching at, or superficial to the border with layer I. The basal dendrites of all pyramidal types are spiny, thin, short and straight, with extensive branches within the most superficial portion of layer III. The maximal mediolateral expanse of the upper and lower dendritic fields in MEC is around 184 ± 75 *μ*m and is therefore smaller than that of stellate cells. The smooth and thin axons of the pyramidal-like cells originate from the soma, some follow a sinusoidal route within layers II and III, giving off collaterals that distribute in layers I–III [129] with an extend that can be larger than that of the dendritic tree (own unpublished data, Alonso et al., 1993). The distribution of the collaterals is comparable to that of stellate cells, but less profuse [118, 128]. Subtypes of pyramidal-like cells have been described including neurons with an obliquely oriented soma and dendrites, called horizontal pyramidal neurons that are mainly located in the superficial part of layer II [122].

Another pyramidal cell type described in LEC has a very thick and sparsely or nonspiny apical dendrite, which branches in layer II. Thin apical dendritic tufts reach layer I. The apical dendrite is not as frequently tilted as in MEC pyramidal neurons [127]. The neurons have thin sparsely spiny basal dendrites and an axon that has extensive collaterals within layers I–III with many varicosities. The main axon of these cells cannot always be followed until the angular bundle but only up to layer III [127, 130].

Interneurons within layer II are described as MPNs, bipolar, basket, and chandelier cells. MPNs have polygonal, fusiform, or round cell bodies with multiple, sparsely spiny dendrites, extending in all directions, reaching layer I and deep into layer III. It has been described that the axons of MPNs travel to the white matter but also form local synapses within layer II [127, 131]. Morphologically they seem to be comparable to stellate cells within the MEC but there are electrophysiological differences. The family of MPNs contains VIP, substance-P, CCK, SOM, ENK, or GABA and in the LEC also NPY [117]. The short-axis cylinder cells in layer III described by Lorento de Nó are comparable to these MPNs [29].

Sparsely spiny horizontal bipolar cells although considered to be local/interneurons project to the hippocampus [115, 119, 131]. The soma is located in layer II at the border to layer I. The dendrites are oriented horizontally along the border between layers I and II [131]. Vertically orientated bipolar cells have a spindle shaped perikaryon continuing into one smooth thin ascending and one descending primary dendrite that branch into thinner dendrites more distally [108]. CR, VIP, and the corticotrophin releasing factor (CRF) have been found in subpopulations of bipolar cells. In the LEC also ENK, CCK and NPY might be present in this class of neurons [115, 117, 119, 131].

Fast spiking basket-like cells have small spherical cell bodies with sparsely spiny dendrites that often ramify into layer I. The extensive axonal arbor is mainly confined to layer II. They form basket-like complexes mainly around the soma of other cells, preferably forming symmetric, inhibitory synapses with stellate or pyramidal cells [117, 132]. Basket cells are known to contain GAD and maybe CCK. Throughout the EC, PV positive axons have been found that form symmetric synapses with principal neurons in layers II and III. These terminals have a basket-like axosomatic configuration. Therefore, it is suggested that basket cells in the whole EC contain PV [117, 132, 133].

Chandelier or axo-axonic cells are characterized by vertical aggregations of axonal boutons, called candles, which preferably are located superficial to the cell body. The somata of chandelier cells are medium sized with different shapes. The almost nonspiny, poorly ramifying dendrites originate from the basal and apical poles of the somata, displaying a bipolar or bitufted arbor that often stays within layer II/III. Vertical chandelier cells that are restricted to MEC issue a vertically oriented axonal tree that is around 200–300 *μ*m wide and 300–450 *μ*m high with the main axonal branch dividing into several collaterals that form the characteristic vertical aggregations within the upper portion of layer II/III [134]. Horizontally organized chandelier cells are located in the MEC and LEC, and their axonal plexi are smaller (250–350 *μ*m wide and 100–200 *μ*m high) than that of vertically oriented chandelier cells. Chandelier cells are GABAergic, often PV-positive and form symmetric contacts with initial axon segments of principal cells [135–138].

**Layer III**

MEC and LEC layer III pyramidal neurons have comparable morphological as well as electrophysiological characteristics [28, 29, 127]. According to some authors, in MEC an anatomical distinction can be made between spiny and nonspiny pyramidal cells [114]. In the LEC, only sparsely spinous pyramidal cells exist that belong to the spiny pyramidal group [127]. The somata of spiny pyramidal cells (SPCs), which are located throughout layer III, give rise to a prominent apical dendrite that bifurcates, become spiny afterwards, and branch extensively. The spiny basal dendrites spread further in the horizontal direction 389 ± 36 *μ*m compared to the vertical direction 203 ± 31 *μ*m, allowing for widespread local connectivity [139]. Apical and basal dendrites together lead to a mean vertical extent of 410 ± 23 *μ*m and a horizontal extent of 312 ± 37 *μ*m. The main axon projects via the angular bundle to the subiculum [139]. Some axonal collaterals spread within layers III and II but also in the lamina dissecans and layer V, occasionally with a broader horizontal extent than the dendritic tree [110, 139, 140].

Nonspiny pyramidal cells (NSPCs), also called type 2 cells [139], have triangular to spherically shaped somata of different sizes. The nonspiny apical dendrite that, compared to SPC, branches significantly closer to the soma also branches frequently in the superficial layers, finally reaching the pia [139, 141]. The vertical dendritic extent of these neurons is comparable to that of SPCs, whereas the horizontal extent, specifically of the basal dendrites, is less [139]. NSPCs thus have a more circular basal dendritic tree around the soma than SPC. The axons of NSPCs travel towards the angular bundle. Collaterals leave the main axon close to the soma and may remain within the corresponding cell layer and/or distribute over all other layers of the entorhinal cortex [139]. The collaterals, which travel towards the superficial layers, sometimes form a net over the entire own dendritic extent and occasionally extending over an even larger domain [110, 139].

Layer III also contains stellate cells, in particular in the upper part of the layer. The somata of these neurons are elongated, polygonal, or spherical. Cells belonging to the latter subgroup sometimes have evenly distributed spiny dendrites around the somata, whereas others have one or two spiny basal dendrites and a variety of ascending dendrites that branch in layer I. The axons reach the white matter, and collaterals are formed in layer III and the lamina dissecans [114].

Also located within layer III are principal MPN somata. These MPNs are either small and spherical, with laterally extending dendrites, or they are large. The largest MPNs are located in the outer half of layer III of the LEC with a conspicuous spatial lateral separation (500 *μ*m) between each cell body. The cell body of large MPNs is 15–18 *μ*m in diameter with multiple sparsely spiny dendrites that elongate in all directions showing moderate branching. The thickest dendrites face towards the superficial layers whereas the thinner ones radiate laterally towards the deep layers. The axons of MPNs reach the hippocampus via the white matter with collaterals distributed in the vicinity of the parent cell soma [114].

Multipolar local circuit neurons, mainly described in MEC, are characterized by wide-ranging apical dendrites that reach the cortical surface, multiple compact basal dendrites, and a prominent axonal arborization. The axon reaches layers I to III but rarely extends into the lamina dissecans or superficial layer V [139]. At least subgroups of MPNs contain GABA, CCK, SOM, substance-P and very rarely SRIF, VIP or ENK [113, 116, 142]. Another subgroup of inhibitory MPNs has sparsely spiny dendrites that extend with their multipolar dendritic arbor towards deep layers instead of superficial layers. In addition, these neurons have an axon extending locally with some collaterals projecting to and reaching layer I [143].

Interneurons resembling pyramidal cells, the so-called pyramidal looking interneurons (PLIs) have also been described as Type 3-(Gloveli) or Type 1-(Kumar) cells [139, 143]. PLIs have a pyramidal shaped cell body and non-spiny basal and apical dendrites branch extensively, forming a dense local network in superficial layers with a circular appearance [139]. The apical dendrites often do not reach the pia and have a vertical dendritic extent of 347 ± 73 *μ*m and a horizontal extend of 269 ± 98 *μ*m. The basal dendrites extend horizontally comparable to that of the apical dendrites. PLIs have a dense axonal plexus in the local vicinity surrounding the cell body, and extending superficially into layer II [143].

Bipolar cells have been described in layer III of MEC and LEC. They have a spindle-like perikaryon with one ascending and one descending smooth, thin and sometimes long dendrite. The ascending dendritic collaterals traverse throughout layer II, reaching layer I. The extent of the descending dendrites has not been described yet. The axon arises from the primary descending dendrite and extends into layer III and the lamina dissecans, deep to the parent cell body [108]. At least a subpopulation of bipolar cells is known to contain VIP or CR. The latter are more common in LEC than in MEC [108, 109].

**Lamina dissecans (layer IV)**

Occasionally, pyramidal-shaped neurons are located in the lamina dissecans, at the borders to layers III and V. These neurons have the morphological and physiological properties of either layer III or layer V pyramidal neurons, respectively (own unpublished data).

Furthermore, bipolar cells, whose dendrites grow horizontally instead of vertically to the pial surface, with axonal collaterals that can travel towards superficial layer III and deep layers, have been found in the lamina dissecans (unpublished data). It has been shown that bipolar cells might contain VIP, CCK, and CRF [117].

**Layer V**

There is no difference between layer V principal neurons in LEC and MEC [144, 145]. The apical dendrites as well as axon collaterals often travel towards superficial layers, sometimes even reaching the pial surface. The basal dendritic tree spreads mainly within deep layers. The main axon travels towards the angular bundle and the subiculum [144, 145]. In general, layer V consists of large pyramidal cells located immediately below the lamina dissecans, while the deeper part of layer V contains smaller cells. The somata of larger pyramidal cells can have different forms. Usually pyramidal formed somata are observed but sometimes also star-shaped cell bodies can be seen. All large pyramidal cells have one distinct large and spiny apical dendrite that often branches close to the soma with the main dendrite reaching the pial surface after branching into a tuft in superficial layers II and I. In case of large pyramidal neurons, spines occur on the dendrites after the first or second bifurcation [120]. The basal dendrites are thinner compared to the apical dendrites and can extent profusely in all directions within layers V and VI [146]. Compared to large pyramidal cells, small neurons have more basal dendrites that are also more densely occupied with spines. These basal dendrites of these smaller cells also extent further in the deep layers. The main axon of the pyramidal cells travels towards the angular bundle, eventually reaching the dentate gyrus via the subiculum [146]. Collaterals of these axons also split within layer V, forming collaterals which travel toward the lamina dissecans, reaching the vicinity of the soma [144, 145]. Some collaterals also travel towards superficial layers [110]. Some pyramidal cells in entorhinal cortex layer V contain SOM [117].

A second principal cell type described in layer V is generally referred to as a type of horizontal cell [29, 120, 144, 145]. Somata of these cells are polygonal rather than pyramidal in shape. A distinct, sparsely spiny, apical dendrite extends to the pial surface, branching extensively in layer I up to the lamina dissecans. In MEC, in contrast to LEC, the primary apical dendrite is not thicker than the other dendrites but is spinier. The characteristic, slightly spiny basal dendritic plexus extents horizontally sometimes up to 1mm from the soma within layers V and VI. Axons of horizontal cells travel to the angular bundle, giving off branches into layers V and VI [110, 144, 145].

A third type of principal neurons is polymorphic MPNs [144–146]. The somata of these cells are spherical to slightly pyramidal with average diameters of 13–24 *μ*m. Instead of having a prominent apical dendrite, these neurons have a multipolar spiny dendritic arborization that extents for long distances in all directions some even into the subiculum ([144–146]; own unpublished data). The axon branches within layer V but reaches the angular bundle and travels through the subiculum, finally reaching the dentate gyrus [146]. Members of the family of MPNs can express PV, SOM, NPY, and substance-P [116, 117].

Fusiform cells that project to the hippocampus were found in superficial layer V [120]. They also have a single ascending dendritic tree that sometimes even reaches the pia and one descending dendritic tree. The axon spreads locally but the main axon projects towards the hippocampus. Fusiform neurons can contain CR [108, 120].

Superficial layer V further harbours bipolar cells with a spindle-like soma having an average diameter along the short axis of around 12 *μ*m [108, 120]. Dendrites originate from the apical and basal poles of the spindle shaped cell body. Except close to the soma, the dendrites are spiny and extend from the soma to the subiculum in one direction and to layer I in the other direction, extensively branching in layers II and I [120]. However, most dendrites are found within the deep layers. The main axon travels towards deep layers and perforates the subiculum, reaching the dentate gyrus [146]. Globular cells have very spiny and highly branched dendrites, originating radially from the soma [120]. Somata have a size of 19.5 *μ*m and up to 12 dendrites that branch within layers III–V. The axon projects towards the angular bundle and within the layer V ([120] own unpublished data). It has been described that multipolar but not explicitly globular cells in the deep layers of the entorhinal cortex might contain SOM, substance-P, NPY, and GABA [117].

**Layer VI**

The multilaminated layer VI borders the white matter. MPNs are located throughout layer VI. They have a spherical soma with a diameter of approximately 14 *μ*m. The spiny dendrites have multiple swellings and extend mainly within layer VI, parallel to the layering. The dendrites also extend towards the angular bundle and rarely to layer III [120]. We found MPNs with basal dendrites with no apical dendrite that surround the soma facing all directions. The axons and collaterals reach the subiculum, whereas other collaterals sometimes reach the superficial layers (own unpublished data).

The somata of classical pyramidal cells in the MEC are medium sized. Pyramidal cells in the LEC have not been described yet. The difference compared to layer V or III pyramidal cells is that the dominant dendrite does not always travel radially towards superficial layers but also either horizontally within layers VI and V or descends towards the angular bundle and the subiculum (own unpublished data). The basal dendrites and the widely spreading collaterals spread within layers VI and V. The axons of pyramidal cells travel towards the angular bundle and subiculum as well as towards superficial layers. Their axon collaterals are located within layers V, VI, the angular bundle, and the subiculum [110].

In conclusion, there are differences between cell types and the distribution of cell types in LEC and MEC (see Figure 2). In layers I and II, the differences between cell morphology and electrophysiology in the LEC compared to the MEC are more prominent than in layers III and V. We know for example that different subtypes of layer I MPNs neurons show a different distribution within layer I. The same holds for chandelier and basket cells in layer II. In addition, there are major differences in the distribution of for example PV- and CR-positive neurons and neuropil. This suggests that LEC and MEC are different with respect to the types of interneurons present. Furthermore, layer II principal neurons in LEC and MEC do not only have a somewhat different morphology but differ also electrophysiologically. Taken together these findings might be an indication that the microcircuits within layers I and II in the LEC and MEC are different. Layer III and V principal neurons of both LEC and MEC are more comparable regarding morphology as well as the electrophysiological properties. Having said this, we need to be aware of the fact that our understanding of the different cell types in the entorhinal cortex and how they are wired together is still rather fragmentary.

### 4.2. Intrinsic organization

The entorhinal cortex contains a substantial system of associational connections that are best described at two different levels. The first is that in all species studied, intraentorhinal fibers are organized in a limited number (generally three) of rostrocaudally oriented bands. Connections that link different transverse (or mediolateral) regions of the entorhinal cortex, thus providing connectivity between these bands, are rather sparse [147–151]. The associational connections within these bands originate in both superficial and deep layers. Results of anatomical tracing experiments have provided convincing evidence that projections originating from layers II and III tend to terminate mainly in the superficial layers, whereas projections originating from deep layers terminate both in the deep and superficial layers. The finding of rather extensive superficial to superficial connectivity seems at odds with results suggesting that there is only sparse collateral innervation among layer II principal cells but see Kumar and Buckmaster [143], who showed layer II to layer II excitatory connectivity with an up to 500 *μ*m distance, and inhibitory connectivity (see also Figure 2). Among layer III principal cells, collateral innervation is more common [152]. One naturally has to take the nature of these local connections into account, and the anatomical results [147–149] do not indicate whether we are dealing with excitatory connections among principal cells or connections of principal cells with putative inhibitory interneurons or even with excitatory local neurons [108]. For example, the pyramidal-like interneurons in layer III or the multipolar interneuron at the border between layers II and III (see Figure 2) is likely candidates to contribute to these intrinsic associative networks, but this remains to be established.

The overall organization of the longitudinal intrinsic connections is best considered in relation to the organization of the reciprocal entorhinal connections with the hippocampal formation. Interconnected portions of the LEC and MEC close to the rhinal fissure, in rats referred to as the dorsolateral band of entorhinal cortex, are connected to the dorsal (nonprimate) or posterior (primate) part of the hippocampal formation (see Figures 1(c), 1(d)). Interconnected cells in the intermediate band, encompassing again parts of both LEC and MEC, connect to the intermediate hippocampal formation, whereas the most medially interconnected band of entorhinal cortex is mainly connected to the ventral (nonprimate) or anterior (primate) hippocampal formation. Cells located in each of these entorhinal bands thus give rise to associational connections to other cells in the same region, but not in any substantial way to portions of the entorhinal cortex that are connected with other levels of the hippocampal formation. Thus, the associational connections seem to be organized to integrate all of the information that targets a particular portion of the entorhinal cortex, and that portion of entorhinal cortex interacts selectively with a particular longitudinal level of the hippocampal formation [2, 147, 153, 154]. This implies that at the level of the entorhinal cortex integration across input modalities may occur and this is in line with reports that in the monkey entorhinal cortex, single neurons apparently respond to different types of sensory inputs [155]. It is still an open question whether these longitudinally organized associative intrinsic networks really support association between the two sets of inputs that reach MEC and LEC, respectively. It is also not known whether this network originates partially or completely from the same neurons that contribute to the more focal intrinsic connectivity that will be described subsequently.

**Figure 3:**
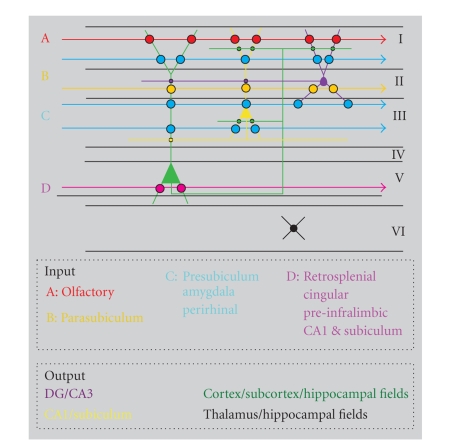
Schematic representation of laminar distribution and synaptic interactions between inputs and principle cells of the entorhinal cortex. Different inputs are represented by color-coded arrows; position of the arrows indicates the main laminar distribution. Circles indicate putative synaptic contacts between inputs and principle cells. Main output connectivity of principle cells is indicated as well. The figure emphasizes the integrative capacity of layer V cells.

The second organizational level deals with the local connectivity within and among layers of more restricted portions of the entorhinal cortex. As we know from the studies summarized above, neurons in different layers have very different inter- and intralaminar connectional patterns that include axon collaterals confined to the parent cell layer or spanning several layers. But not only the axonal distribution is of importance, the dendritic trees may also play an essential role in that they either span several layers or are more restricted to the parent cell layer. Although detailed information for quite a few of neuronal types in the entorhinal cortex is still lacking, it is safe to say that the entorhinal network, on the basis of its neuronal composition alone, cannot be properly described in terms of superficial and deep layers as more or less independent layers. All this may not come as a surprise since comparable concepts have been described with respect to the organization of the neocortex [156]. This second level of intrinsic organization has not yet been seriously incorporated into our working concept about entorhinal cortex. This is essential however in order to properly understand how inputs to the entorhinal cortex will be processed by the entorhinal network and what the eventual information is that will be conveyed to the hippocampal formation on the one hand and to other cortical and subcortical areas on the other hand. The following paragraphs will provide a description of recent most salient findings that may be related to this second level of the entorhinal intrinsic organization (see Figure 3).

One important anatomical observation already reported by Cajal [1, 28] is that neurons in the deep layers are connected to superficial layers by way of axonal projections ([144, 145, 147–149, 157]; see also Figure 2: the small and large pyramidal cells in layer V of both LEC and MEC). These anatomical findings have been corroborated in a number of functional studies [158–162]. Recently, these connections have been studied in more detail in the rat with respect to projections from the subiculum, using both anatomical and electrophysiological techniques [50, 51, 157, 163]. The majority of the axons from deep to superficial layers are likely excitatory and target both interneurons as well as principal neurons in almost equal percentages [157]. This thus constitutes the substrate for powerful excitation as well as feedforward inhibition to neurons in the superficial layers. Stimulation of the subiculum in vivo, not only resulted in population activity in layers II and III of the entorhinal cortex, but subsequently activated the dentate gyrus and CA1 [50, 163]. Moreover, the transfer of activity from layer II to DG and from layer III to CA1 depended on the anesthetic used, suggesting that two functionally distinct parallel reentrant routes exist in the entorhinal-hippocampal system. Although it has not yet been established in freely moving animals whether these two parallel pathways function as separately controlled inputs to DG/CA3 on the one hand and CA1/subiculum on the other hand, the findings are of interest in relation to recently published ideas that DG, together with CA3, is preferentially involved in pattern separation whereas CA1 might be more relevant for pattern completion processes [164–167]. It should be mentioned here that there is also convincing anatomical as well as electrophysiological evidence supporting the existence of connections between cells in layers III and II [114, 148, 149, 168–171] suggesting that they may function in concert as well.

As illustrated in Figure 2, all layers of both LEC and MEC, with the possible exception of layer VI where details are still lacking, contain neurons, mainly of the pyramidal type, with an apical dendrite that extends all the way up to layer I, quite often forming a dendritic tuft in layers II and I. This is a feature that is strikingly similar to what has been reported for the neocortex. Although the functional significance of this general pattern is still poorly understood, the commonality of it, even in evolutionary older parts of the cortex, must be an indication for its significance. In the neocortex, layer I is a main recipient of feedback projections and inputs from subcortical structures [156]. In contrast, in the entorhinal cortex, like in the hippocampus, layer I constitutes a major input layer; for example, the densest innervations from olfactory portions of the cortex, including the olfactory bulb, terminate in layer I [10, 111, 172, 173]. Likewise, in quite a few instances, inputs to the entorhinal cortex that densely terminate in layers II, III, or V have a component to the deep portion of layer I as well [2, 72]. In addition, the apical dendrites of entorhinal neurons not only receive synaptic inputs at their tufts in layer I, but in case of neurons in layer V of MEC, we have shown that they are among the postsynaptic targets of projections from the presubiculum [174]. Note that presubicular inputs to MEC, like those from the perirhinal cortex and some nuclei of the amygdaloid complex, densely terminate in layers III and deep I, almost avoiding layers II and V. Presubicular fibers contact neurons in layer III, and do so with a high density [175, 176]. Recent electrophysiological in vitro and in vivo data have corroborated that presubicular fibers synapse onto neurons in layers III and V, but also onto neurons in layer II [170, 171]. No data are available with respect to perirhinal and amygdale inputs to LEC, that show a similar laminar distribution, but in view of the overall similarity of the networks and cell types in both entorhinal areas, it is likely that for example inputs from perirhinal cortex and amygdale target neurons in layers II, III, and V. Data on inputs that specifically distribute to layer II of MEC, and to a lesser extent of LEC, such as fibers that originate in the parasubiculum, are not available. The potential for functionally relevant interaction between neurons in the deep layers of the entorhinal cortex and superficially terminating inputs has yet another dimension. It has been argued that hippocampal output that leads to firing of cells in layer V of the entorhinal cortex may result in back propagation into the superficial layers, along the dendrites of layer V cells. Back propagation has been documented in the neocortex and in CA1 and may occur in the entorhinal cortex as well. Back-propagating action potentials may increase the influence exerted by inputs to distal portions of the dendrite [163]. This combination of distally terminating inputs from local axon collaterals in layers II and III and back propagation along dendrites of layer V cells provides the most likely substrate for observations that activation of superficial entorhinal layers may lead to subsequent activation of deep layers of entorhinal cortex [158, 159]. Although axons of layers II and III occasionally send a collateral into deep layers of entorhinal cortex ([127]; own observations, illustrated in Figure 2), the overall direct connectivity from superficial to deep layers is rather sparse and therefore may not be sufficient to mediate this rather strong superficial to deep activation.

What then is the functional relevance of inputs from for example the medial prefrontal, cingular, and retrosplenial areas? Afferents from these areas preferentially, and in some instances even exclusively, terminate in the deep layers of the entorhinal cortex. Note that in the monkey, however, it has recently been reported that projections from the retrosplenial cortex densely innervate entorhinal layer I [78]. Do inputs to the deep layers of the entorhinal cortex modulate the transfer of hippocampal output to the cortex, interact with the integrative capacities of the entorhinal network, or both? These questions are relevant not only for our understanding of the functional relevance of the entorhinal cortex in relation to functions of the hippocampus but also since these cortical areas form part of the default mode network, implicated in higher-order cognitive functions [177–179].

## 5. PERSPECTIVES

The functional relevance of the organization of networks in the brain is often interpreted on the basis of a surprisingly restricted point of view. Debates on the functional organization of the hippocampal formation have been strongly influenced by the idea that the prevailing hippocampal circuitry is unidirectional. With regards to the entorhinal cortex, the breakthrough discovery, that deep entorhinal layers receive hippocampal output from CA1 and the subiculum on the one hand, and that these same layers are the origin of strong cortical projections, has biased our view towards the rather simple concept that the deep layers mediate hippocampal-to-cortical connectivity, similar to superficial layers providing the way in for cortical inputs to the hippocampal formation. If the entorhinal cortex is such an important hub, similar to the central station of a large city, and that is what all data seem to converge on, it is quite likely that it serves yet another role. In addition to serving simply to get into the city or leave the city, the station also provides the powerful potential for new interactions between and among incoming and outgoing people. This potential for “new” interactions has been grossly neglected in case of the entorhinal cortex. The potential of the entorhinal cortex to act as an interactive hub, contributing essentially to the functions of the cortico-hippocampal system instead of just transferring information, has been underscored not only by the recent finding of the unique spatial firing properties of grid cells in the entorhinal cortex [7–9], but also by reports that the spatial firing properties of CA1 cells likely depend on inputs from the entorhinal cortex [17–19]. More in particular, the findings that spatially tuned neurons are present in all layers of MEC, and that a clear relationship is apparent between closely associated portions of MEC across layers underscore the concept of the entorhinal cortex as an important higher-order association cortex where understanding the interactions between the layers will provide us the key into its functional relevance [7, 20].

Similar to the yet unresolved mystery of the relevance of cortical inputs to deep layers of the entorhinal cortex, it remains to be established what the functional relevance is of LEC. The data summarized above indicate that with the exception of neurons in layer II, it is likely that both LEC and MEC are largely similar with respect to their intrinsic wiring, both in terms of neuronal elements that comprise the nodal points of the network as well as with respect to how these are wired together (see Figures 2, 3, [20]). What then accounts for the strikingly different features of LEC and MEC when spatially modulated neuronal firing is concerned? Most likely, differences in input and output characteristics will set the scene as eloquently summarized recently [5]. However, how convincing this may look, it may not be the complete story. Additional differences in modulatory connectivity from not only the septal complex, but also from the raphe nuclei, the ventral tegmental area, and locus coeruleus may turn out to be most relevant. Unfortunately, with the partial exception of inputs from the medial septum, very little detailed information is available regarding these inputs in terms of their overall distribution and topography in relation to both extrinsic and intrinsic wiring of the entorhinal cortex. Furthermore, detailed information of the postsynaptic targets of these modulatory inputs is largely missing. One final approach to further our understanding of the entorhinal cortex may be to make use of the striking involvement of the entorhinal cortex in an impressive list of brain diseases [35] and to focus on alterations in the circuitry that likely occur during development, ageing, and disease and their effect on entorhinal functioning.
